# Supplementary figures and images for: Pre-transplant residual diuresis and oxalic acid concentration influence kidney graft survival
Source: PLoS One. 2025 May 16;20(5):e0322516. doi: 10.1371/journal.pone.0322516 (PMC12083809; doi:10.1371/journal.pone.0322516)

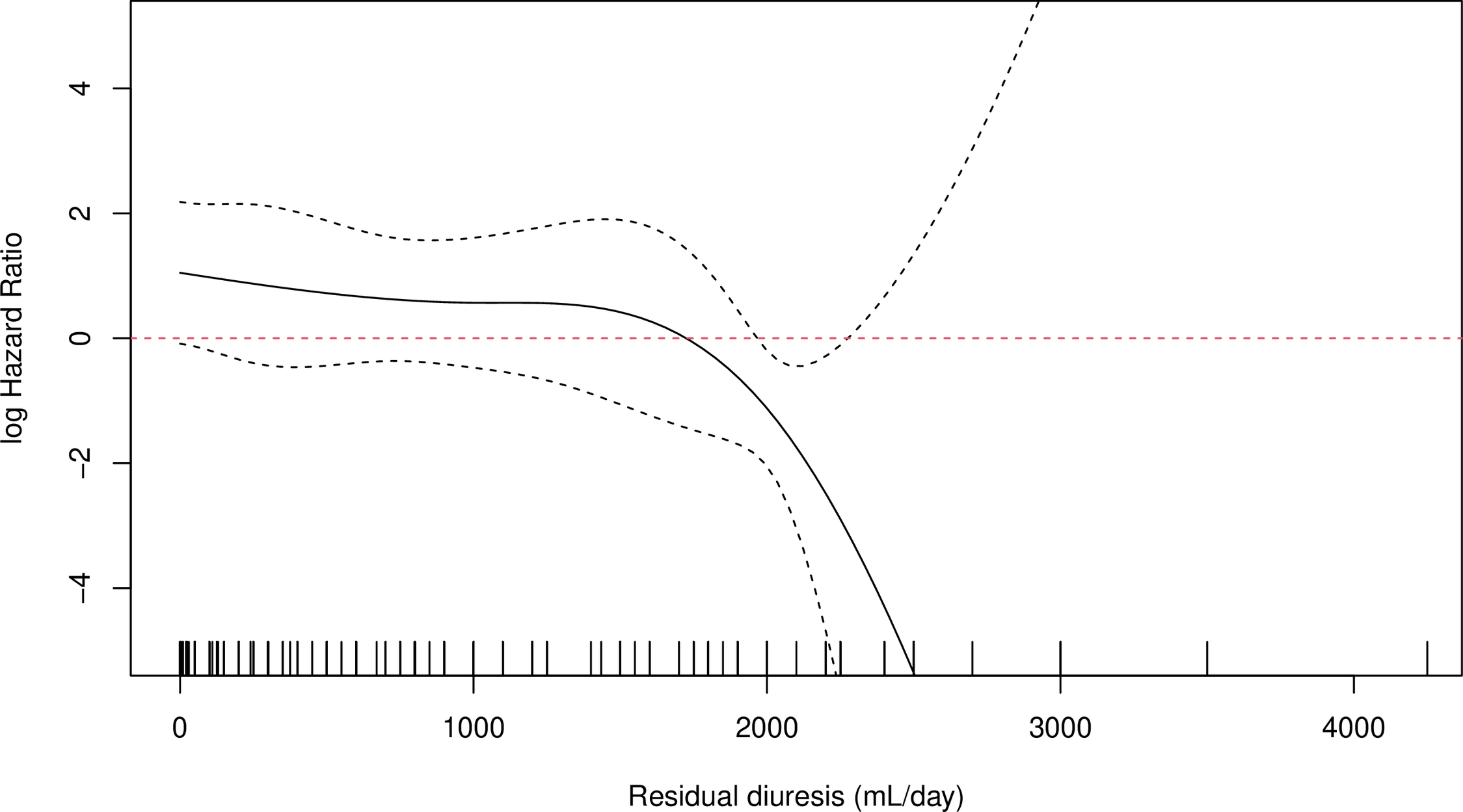

Supplement: S1 Fig — Recipients of a living donor kidney. Donor age is set to 58 years (median age), oxalic acid concentration is set to 33.2 µmol/l (median). Median and IQR of residual diuresis concentration are shown as vertical lines. The small vertical lines above the X-axis represent the observed residual diuresis volumes. The influence of residual diuresis on graft failure risk censored for death becomes significant when the confidence interval exceeds zero (dotted lines). (TIF) [file pone.0322516.s004.tif]
