# Supplementary material for: Pre-transplant residual diuresis and oxalic acid concentration influence kidney graft survival
Source: PLoS One. 2025 May 16;20(5):e0322516. doi: 10.1371/journal.pone.0322516 (PMC12083809; doi:10.1371/journal.pone.0322516)
Supplement: Attachment — Submitted filename: Response to Reviewers.docx [file pone.0322516.s007.docx]

Comments from PLOS Editorial Office:

Please disregard the comments made by reviewer 1, who submitted comments for a different manuscript. The Academic Editor's decision is based on the concerns raised by reviewers 2 and 3.


[Note: HTML markup is below. Please do not edit.]

Reviewers' comments:

Reviewer's Responses to Questions

**Comments to the Author**

1. Is the manuscript technically sound, and do the data support the conclusions?

The manuscript must describe a technically sound piece of scientific research with data that supports the conclusions. Experiments must have been conducted rigorously, with appropriate controls, replication, and sample sizes. The conclusions must be drawn appropriately based on the data presented.

Reviewer #1: Yes Not applicable

Reviewer #2: Partly This is a technically sound prospective cohort study. “Experiments, controls, replications” are not applicable to this type of analysis. Population size is very large for this type of research. The conclusions are drawn based on the data presented and literature available.

Reviewer #3: Yes

2. Has the statistical analysis been performed appropriately and rigorously?

Reviewer #1: I Don't Know Not applicable

Reviewer #2: I Don't Know The statistical analysis has been performed under strict supervision of our statistician Pedro Miranda Afonso from the department of Epidemiology and Biostatistics. Because of his involvement in this study he is awarded as fourth author.

Reviewer #3: Yes

3. Have the authors made all data underlying the findings in their manuscript fully available?

The [PLOS Data policy](https://eur01.safelinks.protection.outlook.com/?url=http%3A%2F%2Ftrack.editorialmanager.com%2FCL0%2Fhttp%3A%252F%252Fwww.plosone.org%252Fstatic%252Fpolicies.action%2523sharing%2F1%2F010f0193d28d8a9d-4aca6863-a0fb-43ef-8d5f-f808c9a97b51-000000%2Fz485zyiKoDVRN90rXBIQwjpOIvLlm3KqYVrbTA42jcM%3D189&data=05%7C02%7Cj.roodnat%40erasmusmc.nl%7Cd7ef9d74b4dc4a42900d08dd1e46b191%7C526638ba6af34b0fa532a1a511f4ac80%7C0%7C0%7C638700011183854577%7CUnknown%7CTWFpbGZsb3d8eyJFbXB0eU1hcGkiOnRydWUsIlYiOiIwLjAuMDAwMCIsIlAiOiJXaW4zMiIsIkFOIjoiTWFpbCIsIldUIjoyfQ%3D%3D%7C0%7C%7C%7C&sdata=Ybuy95rcb%2B5t0vwIZNyUrRtyTp%2F7kAackuIg305u4Q0%3D&reserved=0) requires authors to make all data underlying the findings described in their manuscript fully available without restriction, with rare exception (please refer to the Data Availability Statement in the manuscript PDF file). The data should be provided as part of the manuscript or its supporting information, or deposited to a public repository. For example, in addition to summary statistics, the data points behind means, medians and variance measures should be available. If there are restrictions on publicly sharing data—e.g. participant privacy or use of data from a third party—those must be specified.

Reviewer #1: No Not applicable

Reviewer #2: No Data are available on request.

Reviewer #3: No Data are available on request.

4. Is the manuscript presented in an intelligible fashion and written in standard English?

PLOS ONE does not copyedit accepted manuscripts, so the language in submitted articles must be clear, correct, and unambiguous. Any typographical or grammatical errors should be corrected at revision, so please note any specific errors here.

Reviewer #1: Yes Not applicable

Reviewer #2: Yes

Reviewer #3: Yes

5. Review Comments to the Author
Please use the space provided to explain your answers to the questions above. You may also include additional comments for the author, including concerns about dual publication, research ethics, or publication ethics. (Please upload your review as an attachment if it exceeds 20,000 characters)

Reviewer #1: The authors did a retrospective observational study of potential live kidney donors at their centre between 2007 and 2021 to analyse the causes of their decline Not applicable


Abstract
Line 26, please mention the precent of kidney related conditions that led to donor decline
Line 27, please verify what is meant by medically complex donors and what led to change in your practice in view of your centre protocol. Please provide data supporting that as the results showed only a negative slope of eGFR of accepted donors over time
Line 29, change the sentence “decline in estimated glomerular filtration rate (eGFR) of accepted donors” to “accepting donors with lower eGFR”

Methods
Line 61, can you please mention details about your centre including name, city , average annual transplantation rate

Results
Line 151, change “was female” to “ were females”
Line 153, the authors mentioned no difference when comparing female and male first-degree related donors (p=0.66).
However, the table didn’t provide subdivision of first degree related donors into males and females
It is not clear what is the difference between table 1 and 2 regarding donor sex and relation to recipients. The authors compared declined and accepted donors regarding age, sex and relation to recipient so the denominator should be always the number of donors
Line 160, “(p.adj <0.001)” please correct the typo to (p <0.001).
Line 167, please provide more about your centre protocol , what is the age limit for donation
Figure 1A and 1B provide early the same date, you can keep only 1B as precents are provided
Line 214-216 please provide the statistical test used and the p value for comparison between males and females
Line 216-219, please do a statical comparison between different age groups and provide p value
Line 222, figure 3 A its about sex not age. Please correct this typo
Line 224-227 provide the p value for change in slope of mean age, eGFR, and BMI over time
Line 231, the authors said “The regression line represents the change in donor age per day” I think they meant per year to day
Line 232, , the authors said “To interpret the change per year, multiply the slope coefficient by 365” please clarify this point and provide the change in a table to make it easier for the reader

Reviewer #2: Methods:
• The setting of the study is not clearly defined *Settings are in lines 103-125* *(In the manuscript with track-changes). Extra text is added.*
• Blood samples were taken prior to transplantations, but it does not state how long after last RRT session? It is mentioned in the introduction that oxalic acid levels decrease after dialysis and rebound after 48 hours. This will affect the measured level significantly and creates bias by time between last dialysis session and transplantation. This should be mentioned in the study limitations. *Time between last dialysis session and transplantation is a variable we studied (Table 1). The influence was significant in univariable analysis (supplementary table 2), but not significant in multivariable analysis. Of course concentration of oxalic acid is closely related to time between dialysis and transplantation. We were interested in the influence of the concentration of oxalic acid the newly transplanted kidney is exposed to. Though time did not have a significant influence, concentration of oxalic acid did.*
• “In patients scheduled for a graft biopsy within 3 months after transplantation, blood was taken on the day of the biopsy for determination of serum concentrations of oxalic acid and its precursors.” Why was this follow up level compared to eGFR at day 7 in figure 3, rather than to eGFR at time of biopsy. If this was not a protocol biopsy, then these patients were doing a biopsy for an assumed graft dysfunction, that may have affected the results. Then in the results section it is said that: “Almost all concentrations were obtained within 2 weeks after transplantation (median 7 (IQR 5-10) days after transplantation).” If this is the case, then it should be mentioned in the methods. *You are right, extra text is added to the Methods in lines 124-125.*

• There is no reference provided for the organic acid measurement methodology and for the used statistics. *Extra text on organic acid determination is added in lines 136-145.*

*Statistics were performed in SPSS in close collaboration with our statistician as mentioned in the text. The reference Gauthier (38) was added to the text in line 158 regarding the analysis using cubic splines.*

Results:
• Results, tables and figures are disorganized. *See below. Indeed, in line 235-236 figure 4A and 4B is mentioned that should be figure 2A and 2B. However, as currently 2 extra Figures were added, numbers of figures were adapted again.*
• Table 1: unit of some items are not mentioned, e.g. BMI, median IQR in cold ischemia time. *We added the missing items in Table 1.*
• The categorization of the study cohort by residual urine volume and by oxalic acid levels are not clear and should be presented in a table or graph. *It is known from the literature that there is a super saturation threshold concentration of oxalic acid: above this concentration oxalic acid crystallizes/deposits. This threshold is presumed to be within the range of 30-40 µmol/L (line 67-70). Extra text is added. The cubic spline Cox regression analysis indeed showed a non-linear relationship between oxalic acid concentration and graft survival censored for death (lines 208-209 and Figure 3). This is the reason we had to categorize the variable oxalic acid in 2 groups:* *≤60 and >60* *µmol/L. New text is added in line 212-252 to explain our choice. Evidently, measurable damage only occurs at concentrations above 60 µmol/L; above the supposed super saturation threshold of 30-40 µmol/L (lines 343-344).*

*For residual diuresis see answer to the next remark.*
• Were there 3 categories of residual urine volume (<100, 100-1000 and >1000ml/day) or is it < 200 and > 2000 ml/day as in figures 2A and B, which denote the 25th and 75th percentile? The use of different threshold is a bit confusing. *We describe <1000 and >1000 as clinical information, as many dialysis nephrologists are interested in these thresholds.*

*It is correct that in figures 2A and B (now 4A and B) we show the effect of residual diuresis volume of 200 and 2000 ml/day (25th and 75th percentile) on the graft failure risk.*

*We agree that it may be confusing. Information about the thresholds <1000 and >1000 is removed (lines 196-201).*
• How many deaths occurred and were censored? It is mentioned in the patient survival section but should also be mentioned in the “Graft survival censored for death” section. *Numbers of deaths were added to this section in lines 204-205.*
• “The cubic spline Cox regression model showed a linear relationship between residual diuresis and the log-hazard ratio of graft failure censored for death (graph not shown).” Better present it even if in a supplementary file as it is an important outcome *We added the cubic spline as supplementary figure 1A and B..*
• Figure 4A and B are mentioned before figure 3 and are not present in the manuscript, or are they actually 2A and B? *Yes, you are right. Apologies. Changes were made to line 235-236. However, as 2 Figures are added, numbers of Figures changes again.*
• Consider omitting table 2. *We removed table 2 and added it as supplementary table 2*
• As figure 2 compares between living and deceased donor, why not add a table showing the results in each group regarding the oxalic acid levels, residual volume, death censored graft survival and patient survival? *Figure 4 (was 2) not only compares the influence of living versus deceased donor type but also the influence of residual diuresis, and oxalic acid category in a population with the same donor age. Figure 4 is based on table 2: The final model of multivariable analysis. The only variables with significant influence were residual diuresis, oxalic acid category, donor type and donor age. In the multivariable analysis graft survival censored for death and patient survival is corrected for all variables presented in table 1. Extra text is added to the legend of figure 4 and in line 257.*

*A new versions of Table 1 was produced that shows information on living and deceased donor populations.*

• “In patients with a relatively good functioning kidney graft, oxalic acid concentrations had decreased to concentration below the upper limit of normal. Oxalic acid concentrations were still high in patients with a poorly or nonfunctioning graft (Figure 3).” What is meant by “relatively good functioning kidney” and “poorly or nonfunctioning graft”? This statement is too vague. *You can see that in figure 5: on the Y-axis kidney function (eGFR) and on the X-axis oxalic acid concentration. Text was rephrased in lines 290-294 to be more clear.*
• Any cases of oxalate deposition in the biopsy? *Oxalate depositions in the kidney are not part of routine examination in our center. In only 8 patients oxalate depositions in the biopsy were spontaneously reported. However, visible oxalate depositions are not obligatory for oxalic acid toxicity (see ref 26-29).* *Reference 29 is added.*

Were the patients with high post-transplant oxalate levels further investigated? *Patients with high post-transplant oxalic acid concentration were not separately analyzed, but part of the study. However, in line 297-299 we report that though eGFR was not different between the groups at 1 year after transplantation, the number of graft losses was far higher in the population with oxalic acid concentration >60 µmol/L. This is in line with results of graft survival censored for death. Our clinical experience is that all oxalic acid concentrations decrease as soon as there is a functioning kidney graft. Even moderate kidney function results in a decrease of concentrations.*

• “Variables with significant influence in **univariable analysis** were: recipient age, diabetes mellitus, concentrations of glyceric acid, pre transplant cardiac event, C-reactive protein (CRP) at day of transplantation, type of kidney replacement therapy, time on dialysis (months), body mass index (BMI), residual diuresis per day, previous vascular event, donor type, and donor age (data not shown).” Consider adding a supplementary table. *We removed the text in lines 267-270 and added a supplementary table 3 with these data.*

• “At 1 year after transplantation eGFR (estimated Glomerular Filtration Rate) was not significantly different between the patients with a functioning transplant with pre-transplant oxalic acid concentration ≤60 or >60 µmol/L.” Where are the levels and the significance of their comparison? *You are right, we added the information in lines 297-299.*

Discussion:
• The discussion is presumptive at points and over-interpret the study findings and therefore needs to be re-written*. The discussion is adapted based on the criticisms you describe below.*
• “It is conceivable that excretion of large amounts of these products by the newly transplanted kidney may cause toxicity and damage impairing kidney function.” Yes oxalate are tubulotoxic and may cause oxalate nephropathy, but there is no evidence that short term exposure post-transplant in the recovering graft may have a damaging effect? *In our article about DGF in Transplant International (ref 37) we show that the concentration of the direct precursor of oxalic acid, Glyoxylic acid, is associated with DGF. This is a new and revealing finding! Of course there is convincing literature that DGF results in worse long term graft survival. In the present manuscript, we are the first to show that oxalic acid concentration affects long term graft survival as well: Figures 4A and B show a negative effect of residual diuresis and oxalic acid directly post transplantation, but the lines diverge indicating a continuing negative effect on the long term.*

*There is no other transplant related evidence. However, Ethylene glycol poisoning often leads to (native or graft) kidney damage if untreated. Ethylene glycol is not kidney toxic itself, but its metabolites are kidney toxic: glycoaldehyde, glycolic acid, glyoxylic acid and oxalic acid. We showed that the kidney graft is placed in a toxic environment with high levels of glycolic acid, glyoxylic acid and oxalic acid. This comparison is added to the Introduction in lines 84-94. Figure 1 is added to show how ethylene glycol is metabolized to oxalic acid. References 32, 33 and 34 were added*

• “This means that the pre-transplant patients with low or absent residual diuresis, are less vital than those with significant residual diuresis volume.” What is meant by “less vital”?? *See the lines above this part: “ low residual kidney function is associated with serious comorbidities[27, 41-47].” We added extra text in lines 327-329. These differences are also evident in the dialysis population versus the pre-emptive population. We added a supplementary table 1 that shows characteristics in the populations on dialysis versus pre-emptive patients. Apart from lower residual diuresis, there are significant differences between these populations, mostly unfavorable for the dialysis population: higher recipient age; higher BMI; higher CRP; more cardiac events, vascular events and diabetes mellitus; Higher concentrations of oxalic acid and its precursors; less first transplants; far less living donor transplantations (extra lines 183-186).*

• “Sudden excretion of these products by the newly transplanted kidney may cause damage to the kidney transplant.” There is no evidence backing such an assumption, neither in literature nor in the study findings. *This is our hypothesis for which we found proof in our article on DGF in Transplant International (ref 37). Besides, we showed in the present manuscript that oxalic acid significantly influences long term graft survival censored for death. In our reaction above we made the comparison with ethylene glycol poisoning. We included the latter example in the Introduction in lines 84-94.*
• Although residual urine is a suboptimal marker of residual renal function, the role of residual renal function on patient and graft survival should be further discussed. *See line 319: “There are no studies on the influence of residual diuresis on the graft failure risk after kidney transplantation.” Neither are there studies on residual renal function. We added the latter in line 317. Our findings are new!*
• There should be a clear separation of limitations from the conclusion *This is a text issue. I see what you mean. We adapted the text in lines 375-376.*
• “Dietary and pharmacological interventions are defined to ensure optimal native kidney function preserving care[47].” This statement needs to be further discussed. *Extra text was added in lines 378-380. We added an extra reference on incremental transition to dialysis (53).*

• Recommendations on how to preserve residual urine (and renal function) and lower oxalate levels in ESKD patients need to be separately discussed in a paragraph preceding the limitations. *See lines 380-384. With our guideline we were able to reduce plasma oxalic acid concentrations in ESKD patients with non-renal hyperoxaluria with successful transplantation in the majority of patients. We are currently working on an update of our article ref 55. More than 40 non-renal hyperoxaluria patients have been transplanted in the meantime.*

But are recommendations for patients with enteric hyperoxaluria appropriate for other ESKD patients and will they be as effective? *This indeed is one of the subjects of our studies. Our results show a significant relationship between oxalic acid intake and plasma oxalic acid concentration in patients with end stage renal disease. Depending on revisions, the manuscript is accepted for publication in Clinical Nutrition.*
• A concluding paragraph should be added *We added a conclusion*

Reviewer #3: The manuscript is well written and discussing Important issues and the following are few point to be considered by authors to improve the manuscript:
1- Please define Graft failure in the methods section (On which bases you decided that one recipient has graft failure?). *Definition was added in lines 156-157 (In the manuscript with track-changes).*
2- Please clarify when urine was collected for determinations of residual urine especially for HD patients as urine volume differs between dialysis and non dialysis days. *Definitions regarding urine collection were added to lines 113-115.*
3- in Table 1 you mentioned that 20 had hyperoxaluria non renal causes, please clarify the cause of hyperoxaluria in those patients under or above the table. Has any of them had primary hyperoxaluria? *Extra text was added in lines 186-187.*
4- I think the relation between the Oxalic acid and eGFR day 7 after transplantation is better explained as follow: better eGfr lead to better excretion of oxalic acid so lower oxalic acid level not the reverse. *This is correct, Lines 290-294 were adapted.*

5- In your Conclusion you used your results to support pre-impetive transplantation, to do that you need to show us the difference between candidates who were not on dialysis and those who were on dialysis before transplantation regarding levels of oxalic acid and its metabolites , residual urine volume and outcomes (graft survival and patient survival), please add this comparison in result section. *We added a supplementary table 1 to show the differences between the population of pre-emptive patients versus the population of patients on dialysis. Patients not yet on dialysis have higher residual diuresis volumes and lower oxalic acid concentrations. Both variables have a significant influence on graft survival censored for death in our study. Dialysis type itself does not influence graft survival censored for death in our multivariable analysis.*

*A new Figure 2 was added that shows concentrations of oxalic acid and its precursors in the total population, the pre-emptive population and those on dialysis. Extra text was added in lines 188-191.*

*The (univariable) Kaplan Meier analysis shows the difference in graft survival censored for death in the populations on dialysis versus not yet on dialysis. This is a well-known result. We hypothesize that residual diuresis and concentrations of waste products play a role.*

6- figures need better adjustment (they are hazy and not very clear) *We will sent the original figures.*

6. PLOS authors have the option to publish the peer review history of their article ([what does this mean?](https://eur01.safelinks.protection.outlook.com/?url=https%3A%2F%2Ftrack.editorialmanager.com%2FCL0%2Fhttps%3A%252F%252Fjournals.plos.org%252Fplosone%252Fs%252Feditorial-and-peer-review-process%2523loc-peer-review-history%2F1%2F010f0193d28d8a9d-4aca6863-a0fb-43ef-8d5f-f808c9a97b51-000000%2FRho7RvmZ52E0vKYcoUWEmfqTvXsjavVYtycQIFzzNZU%3D189&data=05%7C02%7Cj.roodnat%40erasmusmc.nl%7Cd7ef9d74b4dc4a42900d08dd1e46b191%7C526638ba6af34b0fa532a1a511f4ac80%7C0%7C0%7C638700011183876539%7CUnknown%7CTWFpbGZsb3d8eyJFbXB0eU1hcGkiOnRydWUsIlYiOiIwLjAuMDAwMCIsIlAiOiJXaW4zMiIsIkFOIjoiTWFpbCIsIldUIjoyfQ%3D%3D%7C0%7C%7C%7C&sdata=uDVwighbQ0qs40ILQxH%2FiPmb0KJOCVMUbPVg7qLfXSQ%3D&reserved=0)). If published, this will include your full peer review and any attached files.

If you choose “no”, your identity will remain anonymous but your review may still be made public.

**Do you want your identity to be public for this peer review?** For information about this choice, including consent withdrawal, please see our [Privacy Policy](https://eur01.safelinks.protection.outlook.com/?url=https%3A%2F%2Ftrack.editorialmanager.com%2FCL0%2Fhttps%3A%252F%252Fwww.plos.org%252Fprivacy-policy%2F1%2F010f0193d28d8a9d-4aca6863-a0fb-43ef-8d5f-f808c9a97b51-000000%2Fp4_EZyC3bgIrpFqrPD2eV2rLcDxKEVPscU8XLfHAweY%3D189&data=05%7C02%7Cj.roodnat%40erasmusmc.nl%7Cd7ef9d74b4dc4a42900d08dd1e46b191%7C526638ba6af34b0fa532a1a511f4ac80%7C0%7C0%7C638700011183893605%7CUnknown%7CTWFpbGZsb3d8eyJFbXB0eU1hcGkiOnRydWUsIlYiOiIwLjAuMDAwMCIsIlAiOiJXaW4zMiIsIkFOIjoiTWFpbCIsIldUIjoyfQ%3D%3D%7C0%7C%7C%7C&sdata=82Y0nCvPX0iccJ1YrHb%2FZfhUOlzvz3j6JM5TPa3wikM%3D&reserved=0).

Reviewer #1: No

Reviewer #2: **Yes:** Yasmine Naga

Reviewer #3: **Yes:** Sara T Ibrahim

[NOTE: If reviewer comments were submitted as an attachment file, they will be attached to this email and accessible via the submission site. Please log into your account, locate the manuscript record, and check for the action link "View Attachments". If this link does not appear, there are no attachment files.]

- - - - -
For journal use only: PONEDEC3

*In compliance with data protection regulations, you may request that we remove your personal*
