# Supplementary material for: Pre-transplant residual diuresis and oxalic acid concentration influence kidney graft survival
Source: PLoS One. 2025 May 16;20(5):e0322516. doi: 10.1371/journal.pone.0322516 (PMC12083809; doi:10.1371/journal.pone.0322516)
Supplement: Attachment — Submitted filename: Response_to_reviewers_auresp_2.docx [file pone.0322516.s008.docx]

14-2-2025

PLOS ONE Decision: Revision required [PONE-D-24-50341R1] - [EMID:2259ed41066de6db]

PLOS ONE

**Answers to Reviewers' comments:**

Reviewer's Responses to Questions

**Comments to the Author**

1. If the authors have adequately addressed your comments raised in a previous round of review and you feel that this manuscript is now acceptable for publication, you may indicate that here to bypass the “Comments to the Author” section, enter your conflict of interest statement in the “Confidential to Editor” section, and submit your "Accept" recommendation.

Reviewer #2: All comments have been addressed

Reviewer #3: All comments have been addressed

Reviewer #4: All comments have been addressed

2. Is the manuscript technically sound, and do the data support the conclusions?

The manuscript must describe a technically sound piece of scientific research with data that supports the conclusions. Experiments must have been conducted rigorously, with appropriate controls, replication, and sample sizes. The conclusions must be drawn appropriately based on the data presented.

Reviewer #2: Yes

Reviewer #3: Yes

Reviewer #4: Yes

3. Has the statistical analysis been performed appropriately and rigorously?

Reviewer #2: Yes

Reviewer #3: Yes

Reviewer #4: Yes

4. Have the authors made all data underlying the findings in their manuscript fully available?

The [PLOS Data policy](https://eur01.safelinks.protection.outlook.com/?url=http%3A%2F%2Ftrack.editorialmanager.com%2FCL0%2Fhttp%3A%252F%252Fwww.plosone.org%252Fstatic%252Fpolicies.action%2523sharing%2F1%2F010f01950688e6d7-6cd49f2f-babd-4339-a574-bb8617fd6690-000000%2FWIY_aU0is55qtSD-7B9qMNjtMHDSjmA2gBLzOuPAepk%3D198&data=05%7C02%7Cj.roodnat%40erasmusmc.nl%7C6e8819137d774a811bf108dd4d453c45%7C526638ba6af34b0fa532a1a511f4ac80%7C0%7C0%7C638751682222354258%7CUnknown%7CTWFpbGZsb3d8eyJFbXB0eU1hcGkiOnRydWUsIlYiOiIwLjAuMDAwMCIsIlAiOiJXaW4zMiIsIkFOIjoiTWFpbCIsIldUIjoyfQ%3D%3D%7C0%7C%7C%7C&sdata=2KQF2Mtb9JzcP26IrImkqE%2B8Hi6%2BLCPUe2OyXJGz2Ow%3D&reserved=0) requires authors to make all data underlying the findings described in their manuscript fully available without restriction, with rare exception (please refer to the Data Availability Statement in the manuscript PDF file). The data should be provided as part of the manuscript or its supporting information, or deposited to a public repository. For example, in addition to summary statistics, the data points behind means, medians and variance measures should be available. If there are restrictions on publicly sharing data—e.g. participant privacy or use of data from a third party—those must be specified.

Reviewer #2: Yes

Reviewer #3: Yes

Reviewer #4: Yes

5. Is the manuscript presented in an intelligible fashion and written in standard English?

PLOS ONE does not copyedit accepted manuscripts, so the language in submitted articles must be clear, correct, and unambiguous. Any typographical or grammatical errors should be corrected at revision, so please note any specific errors here.

Reviewer #2: Yes

Reviewer #3: Yes

Reviewer #4: Yes

6. Review Comments to the Author

Please use the space provided to explain your answers to the questions above. You may also include additional comments for the author, including concerns about dual publication, research ethics, or publication ethics. (Please upload your review as an attachment if it exceeds 20,000 characters)

Reviewer #2: Most of the previously mentioned comments were adequately addressed. But a few changes still need to be done.

Introduction:
The introduction is a bit disjointed, and the paragraph need sentence openers to connect them.

*Thank you for your efforts to improve our manuscript. Indeed, the Introduction improved significantly after textual adjustments to improve the connection in lines 60-105.*

Methods:
The organic acid measurement method is not described in detail but us not backed by any reference. Is the methos novel or has it been previously used and described in literature?

*Groningen is the reference lab for clinical chemistry and metabolic diseases for the northern half of the Netherlands since many decades. The method used is a further developed and improved method, based on previous methods since the seventies of the former century. The current method is currently being written into a manuscript for publication.*

*Extra text is added to line 141. The methods, on which the current method is based, are referred in line 144.*

*Extra text on the internal quality control, sensitivity and specificity was added to lines 151-167.*

Results:
“There were 19 patients with enteric hyperoxaluria, one patient with primary hyperoxaluria was included.” Was the diagnosis made before transplantation or after. Did the patient with primary hyperoxaluria receive a liver transplant?

*Indeed an important clinical question. Extra text is added to lines 207-210 on this primary hyperoxaluria patient.*

Always put a comma before “”respectively”

*The text was adapted in lines 215, and 305.*

“In our population, the estimated turning point is at about 60 µmol/L in the recipient population with a living donor and at 70 µmol/L, in the recipient population with a deceased donor kidney” better replace the term turning point. Was it the level at which increased graft survival decreased

*Indeed, above this concentration the influence on graft survival is significant. Turning point was replaced by threshold for the influence on graft survival (line 231-232).*

Reviewer #3: (No Response)

Reviewer #4: I read with interest the revised version of this manuscript regarding the role of residual kidney function in limiting the role of oxalate toxicity after transplant. I only have some minor comments about how to improve the high quality of the manuscript.
- Despite not being reported and standardized as in the present study, we have the same clinical impression about patients with and without residual diuresis; patients without residual diuresis often showed extrarenal deposits of oxalate (e.g., in bone and soft tissues) and frequently experienced DGF, so we performed in some cases post-transplant dialysis in patients with not satisfying diuresis output and combined high oxalate levels. Do the authors have the same experience or suggest some strategies for patients at high risk for tubular toxicity (e.g., belatacept used to reduce combined CNI toxicity as in another clinical setting, see in example 10.1371/journal.pone.0240335)?

*Thank you for your compliment. Your description of extrarenal oxalate deposits and DGF in patients with low residual diuresis is really interesting and would perfectly fit into the experience we describe in the current manuscript. Do you have a protocol for post-transplant dialysis sessions in this population?*

*We do have a guideline for hyperoxaluria patients (primary or enteric) with high oxalic acid plasma concentrations: we try to decrease pre-transplantation oxalic acid concentrations and offer low-threshold daily dialysis sessions from day 1 onwards when urine production is below 1L per day in the first 24 hours after transplantation. Pre-transplant oxalic acid concentrations are determined only on indication: when primary or enteric hyperoxaluria is supposed. This means that this guideline does not hold for the entire transplant population.*

*We describe this guideline in our article from 2017 (reference 55) and are currently working on an update including 41 hyperoxaluria patients.*

*In our center Belatacept is seldom used to enable a decreased dose of CNI. We do not have a protocol for that.*

- At the same time, incremental dialysis and high-volume sessions (e.g., five times/week) in patients on active waiting list with progressive reduction of urinary output were suggested in our center to reduce the oxalate deposition. Have the authors suggested or implemented this strategy based on their results? Please comment on it.

*We do have this guideline, as described above, for patients with non-renal hyperoxaluria (primary or enteric). When pre-transplant oxalic acid concentration is above 60 µmol/L we suggest intensified dialysis sessions in the period before transplantation. It is left to the patient and treating nephrologist to what extent. In patients with a living donor dialysis is intensified (daily sessions) in the week before the transplantation. In patients awaiting a deceased donor offer dialysis can be intensified by switching to night dialysis or as you state: as many hours but in four to five times per week.*

7. PLOS authors have the option to publish the peer review history of their article ([what does this mean?](https://eur01.safelinks.protection.outlook.com/?url=https%3A%2F%2Ftrack.editorialmanager.com%2FCL0%2Fhttps%3A%252F%252Fjournals.plos.org%252Fplosone%252Fs%252Feditorial-and-peer-review-process%2523loc-peer-review-history%2F1%2F010f01950688e6d7-6cd49f2f-babd-4339-a574-bb8617fd6690-000000%2FpRgVRJysCh9hGdtWqc2LDQ4UzBYwb7XwQ7Fu7C-epBs%3D198&data=05%7C02%7Cj.roodnat%40erasmusmc.nl%7C6e8819137d774a811bf108dd4d453c45%7C526638ba6af34b0fa532a1a511f4ac80%7C0%7C0%7C638751682222371859%7CUnknown%7CTWFpbGZsb3d8eyJFbXB0eU1hcGkiOnRydWUsIlYiOiIwLjAuMDAwMCIsIlAiOiJXaW4zMiIsIkFOIjoiTWFpbCIsIldUIjoyfQ%3D%3D%7C0%7C%7C%7C&sdata=3ErAUucbwk0bISqhF5W8vrpCmTHq40lS62LumCTOukU%3D&reserved=0)). If published, this will include your full peer review and any attached files.


If you choose “no”, your identity will remain anonymous but your review may still be made public.


**Do you want your identity to be public for this peer review?** For information about this choice, including consent withdrawal, please see our [Privacy Policy](https://eur01.safelinks.protection.outlook.com/?url=https%3A%2F%2Ftrack.editorialmanager.com%2FCL0%2Fhttps%3A%252F%252Fwww.plos.org%252Fprivacy-policy%2F1%2F010f01950688e6d7-6cd49f2f-babd-4339-a574-bb8617fd6690-000000%2Ff0K0sMKLGq3CLWYHgVm-DTHlxM-l-6BF63n84ntcYx4%3D198&data=05%7C02%7Cj.roodnat%40erasmusmc.nl%7C6e8819137d774a811bf108dd4d453c45%7C526638ba6af34b0fa532a1a511f4ac80%7C0%7C0%7C638751682222389469%7CUnknown%7CTWFpbGZsb3d8eyJFbXB0eU1hcGkiOnRydWUsIlYiOiIwLjAuMDAwMCIsIlAiOiJXaW4zMiIsIkFOIjoiTWFpbCIsIldUIjoyfQ%3D%3D%7C0%7C%7C%7C&sdata=ZwUoxYYZNKNoFFXPsXmViYNJz%2B2nptzOROnQEpTXGfA%3D&reserved=0).

Reviewer #2: **Yes:** Yasmine Naga

Reviewer #3: **Yes:** Sara T Ibrahim

Reviewer #4: No

[NOTE: If reviewer comments were submitted as an attachment file, they will be attached to this email and accessible via the submission site. Please log into your account, locate the manuscript record, and check for the action link "View Attachments". If this link does not appear, there are no attachment files.]

While revising your submission, please upload your figure files to the Preflight Analysis and Conversion Engine (PACE) digital diagnostic tool, [https://pacev2.apexcovantage.com/](https://eur01.safelinks.protection.outlook.com/?url=https%3A%2F%2Ftrack.editorialmanager.com%2FCL0%2Fhttps%3A%252F%252Fpacev2.apexcovantage.com%252F%2F1%2F010f01950688e6d7-6cd49f2f-babd-4339-a574-bb8617fd6690-000000%2FEu4wPQlhFAXn6Wl2hBdVBBZfLC6PS06CV-CzIrpbnFI%3D198&data=05%7C02%7Cj.roodnat%40erasmusmc.nl%7C6e8819137d774a811bf108dd4d453c45%7C526638ba6af34b0fa532a1a511f4ac80%7C0%7C0%7C638751682222406757%7CUnknown%7CTWFpbGZsb3d8eyJFbXB0eU1hcGkiOnRydWUsIlYiOiIwLjAuMDAwMCIsIlAiOiJXaW4zMiIsIkFOIjoiTWFpbCIsIldUIjoyfQ%3D%3D%7C0%7C%7C%7C&sdata=VdSO1EQfjt8i2tQTVORnUIOSDZPuBpgRKh3vHSYs3X8%3D&reserved=0). PACE helps ensure that figures meet PLOS requirements. To use PACE, you must first register as a user. Registration is free. Then, login and navigate to the UPLOAD tab, where you will find detailed instructions on how to use the tool. If you encounter any issues or have any questions when using PACE, please email PLOS at [figures@plos.org](mailto:figures@plos.org). Please note that Supporting Information files do not need this step.

*We successfully used PACE to convert and upload our graphs.*
